# The effect of racial and gender concordance between physicians and patients on the assessment of hospitalist performance: a pilot study

**DOI:** 10.1186/s12913-019-4090-5

**Published:** 2019-04-24

**Authors:** Damian Crawford, Suchitra Paranji, Shalini Chandra, Scott Wright, Flora Kisuule

**Affiliations:** 1 Division of Hospital Medicine, Johns Hopkins Bayview Medical Center, Johns Hopkins University School of Medicine, Baltimore, MD USA; 2 Division of General Internal Medicine, Johns Hopkins Bayview Medical Center, Johns Hopkins University School of Medicine, Baltimore, USA; 3 Johns Hopkins University School of Medicine, Johns Hopkins Bayview Medical Center, 5200 Eastern Avenue, MFL Building West Tower 6th Floor CIMS Suite, Baltimore, MD 21224 USA

**Keywords:** Hospitalization, Patient satisfaction, Gender, Race, Concordance

## Abstract

**Background:**

Lack of racial concordance between physicians and patients has been linked to health disparities and inequities. Studies show that patients prefer physicians who look like them; however, there are too few underrepresented minority physicians in the workforce. Hospitalists are Internal Medicine physicians who specialize in inpatient medicine. At our hospital, hospitalists care for 60% of hospitalized medical patients. We utilized the validated Tool to Assess Inpatient Satisfaction with Care from Hospitalists (TAISCH) to assess the effect of patient-provider race and gender concordance on patients’ assessment of their physician’s performance.

**Methods:**

Four hundred thirty-seven inpatients admitted to the non-teaching hospitalist service, cared for by a unique hospitalist physician for two or more consecutive days, were surveyed using the validated TAISCH instrument. The influence of gender and racial concordance on TAISCH scores for patient - hospitalist pairs were assessed by comparing the specific dyads with the overall mean scores. T-tests were used to compare the means. Generalized estimating equations were used to account for clustering.

**Results:**

Of the 34 hospitalist physicians in the analysis, 20% were African American (AA-non-Hispanic), 15% were Caucasians (non-Hispanic) and 65% were in the “other” category. The “other” category consisted of predominantly physicians of South East Asian decent (i.e. Indian subcontinent) and Hispanic. Of the 437 patients, 66% were Caucasians, and 32% were AA. The overall mean TAISCH score, as these 437 patients assessed their hospitalist provider was 3.8 (se = 0.60). The highest mean TAISCH score was for the Caucasian provider-AA patient dyads at 4.2 (se = 0.21, *p* = 0.05 compared to overall mean). The lowest mean TAISCH score was 3.5 (se = 0.14) seen in the AA provider/AA patient dyads, significantly lower than the overall mean (*p* = 0.013). There were no statistically significant differences noted between mean TAISCH scores of gender and racially concordant versus discordant doctor-patient dyads (all p’s > 0.05).

**Conclusions:**

In the inpatient setting, it appears as if neither race nor gender concordance with the provider affects a patient’s assessment of a hospitalist’s performance.

## Background

In the United States, there are differences in clinically significant health outcomes based on race and gender [1]. Racial disparities, in particular, are notable in the care of cancer, pain, and diabetes management [2]. Biases have been linked to inequities in the treatment of cardiovascular diseases, translating into substantive differences in mortality rates across races [3]. Minority patients perceive higher rates of discrimination in healthcare compared to Caucasian patients [4]. As such, it is not surprising that in some studies minority patients expressed preferences to be cared for by providers of their same race/ethnicity and also rate their visits with race-concordant providers more positively [5]. Racial concordance may augment patients’ sense of being understood, and might allow for more trusting connections to develop quickly. In one study, Caucasian patients were 33% more likely to report medical errors when the provider was non-white compared to when there was racial concordance [4]. Physician gender is also believed to influence the patient experience. In some studies, patients of female primary care physicians were more satisfied than those of their male counterparts, even after adjusting for patient characteristics, visit length, and physician practice style [6]. There is also evidence that women report more adherence to mammography screening with a racially concordant physician [7, 8].

Patient perception of care, in this era of value-based purchasing in healthcare, is linked to enhanced compliance and better clinical outcomes [9]. Such assessment of care is linked to patient satisfaction as a measure of the quality of care provided to the patient [10]. In 2014, we developed and validated a *Tool to Assess Inpatient Satisfaction With Care From Hospitalists* (TAISCH) [11]. Hospital medicine is the fastest growing specialty in the United States. Hospitalists are physicians who specialize in caring for hospitalized patients [12, 13]. The hospitalist field emerged as a response to a combination of forces including primary care providers providing less inpatient care and the need for 24/7 on-site high-value hospital patient care [14]. Hospitals and providers are concerned about the validity of the measures currently used to obtain patient satisfaction scores. A major issue with these assessments is that multiple providers are commonly involved in the care of a patient during a hospitalization and attributing a patient satisfaction score to one hospitalist can be problematic. The traditionally mailed surveys are limited in that they (i) ask only a few questions about a doctors’ performance, (ii) are completed long after the hospitalization thus being subject to recall bias, and (iii) are notorious for having extremely low response rates.

TAISCH collects detailed assessment in real-time which ensures that the patient is considering their current hospitalist when answering questions. The instrument was specifically designed to have hospitalized patients comprehensively rate aspects of hospitalists’ performance across 6 domains prioritized by the Society of Hospital Medicine (SHM) including: physician availability, physician concern for patients, physician communication skills, physician courteousness, physician clinical skills, and physician involvement with patient families [11].

Several studies have shown that gender and racial concordance between physicians and patients in the outpatient setting affects patient assessment of physician performance [5, 15–17]. However, there are no studies specifically looking at the effect of race and gender concordance on patients’ perception of hospitalist performance. We conducted this pilot study to determine the effect of physician-patient gender and race concordance on inpatient rating of hospitalists’ performance.

## Methods

### Study design, patients, and setting

This cross-sectional study prospectively collected data from inpatients who were cared for on a general medical, non-teaching unit, at our 426-bed academic medical center during academic years 2012–2013 and 2014–2015. TAISCH was not administered and collected during academic year 2013–2014. A total of 437 patients and the 34 hospitalist physicians who took care of them are included in the study analysis. Patients who were on isolation precautions, non-English speaking, and those with altered mental status or dementia were excluded from the study.

### Data collection

After obtaining consent to participate, a research assistant asked all eligible inpatients who saw a single hospitalist over two consecutive days to identify the physician from a list of physician pictures. Those who could correctly identify their provider were asked to rate their provider across 15 five-point Likert scale items on TAISCH [11] while they were still hospitalized. In addition, patient gender and race data were collected and recorded for all patient-provider dyads. Race was determined by self-identification by both the patients and physicians. The discharge diagnoses among the hospitalized patients were also recorded.

### Data analysis

Descriptive characteristics, including means and standard deviations, were calculated for all variables. TAISCH scores range from 1 to 5, with 5 indicating the highest rating by the patient for the physicians’ performance. The data was analyzed to calculate the mean TAISCH score [for all physicians]. We then explored associations between both gender and racial concordance of the physician-patient dyads by comparing TAISCH scores for specific dyads (including concordant and non-concordant pairs) with the overall mean TAISCH score using t-tests, for this normally distributed data. *P*-values less than 0.05 were considered to be statistically significant. Generalized Estimating Equations (GEE) were used to account for clustering, given the fact that some hospitalists had multiple patient encounters.

The data was analyzed using Stata version 12.0 (StataCorp Inc., College Station, Tx). This study was approved by the Institutional Review Board of Johns Hopkins University NA_00049144.

## Results

Of the 34 hospitalist providers, 7 (20%) were African-American (AA), 5 (15%) were Caucasian, and 22 (65%) were ‘other’. The other category was predominantly physicians who identify as Southeast Asian in origin. Out of the 437 patients that were surveyed, 290 (66%) were Caucasian; 139 (32%) were AA, and 8 (2%) were ‘other’ (neither AA nor Caucasian). The five most common discharge diagnoses among those surveyed during the study period were pneumonia, chronic obstructive pulmonary disease exacerbation, acute kidney failure, alcohol withdrawal, and decompensated heart failure.

The overall mean TAISCH score, as these 437 patients assessed their hospitalist provider was 3.8 (se = 0.60).

### Gender concordance

The mean TAISCH scores for gender concordant and non-concordant doctor-patient dyads ranged from 3.7 to 3.9 (see Table 1). Gender concordant dyads were non-statistically significantly higher than gender discordant pairs (all p’s > 0.05).

**Table 1:** TAISCH Scores Based on Gender Concordance and Discordance

Provider/Patient Dyads	Mean (SE) TAISCH Scores	P-value comparing the specific dyad score to the overall mean^a^	Number of patients	Number of providers
Examining Gender Concordance				
Female/ Female	3.9 (0.1)	0.58	102	15
Female/ Male	3.8 (0.1)	0.89	77	15
Male/ Female	3.7 (0.1)	0.16	143	19
Male/ Male	3.9 (0.1)	0.40	115	17
Examining Racial Concordance				
AA/AA	3.5 (0.1)	0.01	39	7
AA/ Caucasian	3.8 (0.1)	0.76	83	7
Caucasian/ AA	4.2 (0.1)	0.05	17	5
Caucasian/ Caucasian	4.0 (0.1)	0.28	32	5
Other/AA	3.9 (0.1)	0.65	83	19
Other/Caucasian	3.8 (0.1)	0.88	174	22

The categories AA/Other, Other/Other and Caucasian/Other were omitted as the number of patients belonging to the ‘Other’ category constituted of just 1.4% of the total sample

*AA* African American, *Other* Asian and Hispanic

^a^ The overall mean TAISCH score, as these 437 patients assessed their hospitalist provider was 3.8 (0.60)

### Racial concordance

The highest mean TAISCH scores noted for any racial pair was 4.2 (se = 0.21, *p* = 0.05 when compared to the overall mean TAISCH score) in the Caucasian provider-AA patient dyads (see Table 1). The lowest mean TAISCH score of 3.5 (se = 0.14) seen in the AA provider and AA patient dyads, which was significantly lower (*p* = 0.013) than the overall mean TAISCH score.

Analyses with and without GEE to account for potential clustering yielded essentially identical findings, without any changes in the statistical significance of any of the associations described above.

## Discussion

Our study showed no significant statistical enhancement in hospitalists’ assessments by patients attributable to gender or racial concordance in the inpatient setting using TAISCH. In fact, AA patients rated AA hospitalist physicians more sternly. These new data are interesting since the evidence to date indicates that patients have greater satisfaction with racially concordant physicians [16]. The available literature is rich in studies that show that gender and racial concordance between physicians and patients in the outpatient setting positively affects patient assessment of physician performance [15].

The limited impact of gender and racial concordance between patients and doctors on physician assessment in our study could be due to several factors. Hospitalized patients are sicker than their outpatient counterparts and as such they may be intently focused on recovery and less concerned with the race or gender of their provider. In a study from Singapore, illness management was the most important domain of patient satisfaction for patients in the ICU [18]. Furthermore, in the inpatient setting patients are not able to choose their own provider. The provider they are assigned is entirely left to chance and is essentially based on who is working on that day. In the outpatient setting, patients are able to “doctor shop” and choose physicians who they want and believe may be a good fit; some might consider racial and gender concordance if they prioritize these variables and they live in a place where there is a diverse healthcare workforce. An additional reason as to why gender and racial concordance between patient and doctors may be of lesser importance for hospitalized individuals could include the fact that patients interact with many members of the multidisciplinary care team each day, and these teams are usually well diversified in all respects.

In this study, the Caucasian/AA provider/patient dyad had the highest overall scores, while the AA/AA dyad had the lowest mean scores. Prior studies have suggested that African American patients tend to prefer AA providers [5, 15, 19]. However, AA trust may not entirely be based on race; rather communication across cultural and language barriers is known to be highly valued [20]. The Caucasian hospitalists in our group have been caring for a high proportion of AA patients for many years and they have become culturally competent with cultural humility [21]. Further, because Caucasian providers represent the majority of physicians in the country, the status quo, all patients might just feel more comfortable (and appraise them more highly on validated scales) based on familiarity.

There is evidence that culturally competent physicians have a positive effect on patient satisfaction, equity, and on decreasing disparities [22–24]. The existing diversity within our physician group, attributable to our efforts to recruit with attention to equity in gender and race, has fostered the team that is culturally competent and humble. This reality may have mitigated against greater variation in TAISCH scores with respect to racial and gender differences among patient and physicians. Our group culture might have sensitized providers to racial and gender nuances; not only have we recruited providers who ‘buy-into’ this philosophy but we have weeded out providers who did not prioritize these values and expected norms.

There are several limitations to this study that should be considered. First, this study was conducted exclusively with patients at one hospital. Patients’ attitude towards providers might be different depending on prevailing geographical, political, and cultural climates. However, the data was collected from two study periods and this could help mitigate attitudes that would have been popular or trending at the time. Second, approximately 15% of the physicians and 66% of the patients in this study were Caucasian, as compared to 49% of physicians and 61% of the population nationally [25]. Third, our study excluded non-English speakers as this was an exclusion criterion during the validation of the TAISCH tool. Future studies should include this growing demographic to more fully understand the relationships that were studied. Finally, our sample size was relatively small. However, we were able to detect statistically significant differences on the TAISCH instrument across some of the specific provider-patient dyads. Larger studies conducted at multiple institutions will assess the generalizability of the findings noted in this pilot study.

## Conclusion

This study assessed hospitalist performance using the TAISCH instrument, which yields undeniably attributable assessments of providers by patients, and showed that neither gender nor race concordance enhanced the appraisals. These results are contrary to research findings conducted in outpatient settings, that have shown that patient satisfaction is affected racial and gender concordance between patients and providers. Future research examining the effect of racial and gender concordance between physicians and patients, looking at distinct subsets of patients and specific inpatient settings, may enhance our understanding of these matters.

## Abbreviations

AA: African American
FMG: Foreign Medical Graduates
HCAHPS: Hospital Consumer Assessment of Healthcare Providers
SHM: Society of Hospital Medicine
TAISCH: Tool to Assess Inpatient Satisfaction With Care From Hospitalists
